# Beyond self-report: Measuring visual, auditory, and tactile mental imagery using a mental comparison task

**DOI:** 10.3758/s13428-024-02496-z

**Published:** 2024-09-13

**Authors:** Sebastian Paul Suggate

**Affiliations:** Department of Education, University of Regensburg, Regensburg, Germany

**Keywords:** Mental imagery, Imagination, Visual, Tactile, Self-report imagery

## Abstract

Finding a reliable and objective measure of individual differences in mental imagery across sensory modalities is difficult, with measures relying on self-report scales or focusing on one modality alone. Based on the idea that mental imagery involves multimodal sensorimotor simulations, a mental comparison task (MCT) was developed across three studies and tested on adults (*n* = 96, 345, and 448). Analyses examined: (a) the internal consistency of the MCT, (b) whether lexical features of the MCT stimuli (word length and frequency) predicted performance, (c) whether the MCT related to two widely used self-report scales, (d) response latencies and accuracies across the visual, auditory, and tactile modalities, and (e) whether MCT performance was independent of processing speed. The MCT showed evidence of reliability and validity. Responses were fastest and most accurate for the visual modality, followed by the auditory and tactile. However, consistent with the idea that self-report questionnaires index a different aspect of mental imagery, the MCT showed minimal correlations with self-report imagery. Finally, relations between MCT scales remained strong after controlling for processing speed. Findings are discussed in relation to current understanding and measurement of mental imagery.

## Introduction

The existence or experience of mental imagery is inherently enigmatic, yet of fundamental importance to the human mind and experience, weighing into fundamental ideas about conscious experience and the philosophy of mind itself (Kunzendorf, 2021). Some, like the blind and deaf writer Helen Keller, could use linguistic wizardry to conjure rich mental pictures in readers, despite the loss of senses. Others, like the behaviorist John Watson, saw in the idea of mental imagery nothing but sentimentality (Thomas, 2020). Recently, building on initial beginnings (Galton, 1880), research has identified the existence of different profiles of imagery, with some reporting no imagery whatsoever (i.e., aphantasia) and others vast imaginative worlds (i.e., hyperphantasia, Pearson, 2019; Zeman et al., 2015).

In this paper, current measures of mental imagery are reviewed and, it is argued that, although there are a plethora of self-report imagery scales available (Dahm, 2020; Suica et al., 2022), the cupboard is somewhat bare in terms of objective, performance measures. Self-report tasks may be inherently flawed as measures of individual differences in imagery because a participant cannot be expected to judge how good their imagery is compared to someone else’s, whose images they presumably cannot directly perceive (i.e., “can I visualize a zebra better than my friend can?”). One objective measure is the mental comparisons task (MCT, Moyer, 1973), which holds promise for measuring mental imagery in a range of different modalities (e.g., visual, tactile, auditory) and can be administered easily, in a time-efficient manner (e.g., Martzog & Suggate, 2019). However, the MCT requires further testing and development, which is the aim of the current paper.

### Mental imagery

Here, mental imagery is, broadly defined, the ability to experience conscious sensorimotor simulations of events, scenes, and states in the physical absence of these to the senses. Specifically, mental imagery is often associated with the ability to construct, inspect, manipulate, and transform images in the mind’s eye (Kosslyn et al., 1990). Based on previous research (Barsalou, 1999; Brogaard & Gatzia, 2017; Cole et al., 2022; Paivio, 2013; Pearson, 2019), three key findings of mental imagery will be outlined, which help contextualise past, present, and future research. Specifically, mental imagery is (a) multi-modal, (b) utilizes many of the same systems and processes as perception, and (c) shows large individual differences in conscious experience.

However, it is first important to acknowledge that the existence of mental imagery itself is not without controversy. A criticism is that mental imagery is not (always) central to cognition, that it may be an epiphenomenal offshoot (Cole et al., 2022. It has been argued that the content of cognition is fundamentally propositional, abstract and amodal (Pylyshyn, 2002), or at least that it can be much of the time (Paivio, 2013). Indeed, given that it is widely acknowledged that images are constructed, a logical criticism is that if images are constructed from different units of information, then the individual already had the information in the first place. According to this idea, imagery could not logically add more to cognition because it represents nothing new (Cole et al., 2022). On the other hand, it is conceivable that juxtapositions of different images might lead to new insights (e.g., if a sofa or a table were placed in an empty room, which would leave more space in front of the window?).

A further debate revolves around whether the smaller units comprising images are informational/propositional (Kunzendorf, 2021) or perceptual (Barsalou, 1999). Others have argued for both positions, essentially that cognition is stored in two modes, either a propositional and/or a sensorimotor code (Paivio, 2013). Indeed, Pylshyn points out a number of problems with pictorial imagery theories and argues that all previous mental imagery tasks can be solved propositionally (Pylyshyn, 2002). In support of this idea and as outlined, the rapidly expanding research on aphantasia has found very few instances of cognitive deficits, aside from some evidence of reduced facial recognition and autobiographical memory (Cavedon-Taylor, 2022; Dawes et al., 2020, Milton et al., 2021; Zeman et al., 2020).

### Mental imagery is multi-modal

Arguably, and more so in lay-circles, imagery is thought of as being visual, although the stem “imagos” originally meant “likeness” or “copy” in Latin and hence not restricted to a visual image. Perhaps somewhat unfortuitously the condition, where people apparently experience no mental imagery (i.e., aphantasia), is often determined by taking scores on a measure of the vividness of *visual* imagery (Zeman et al., 2015). Despite arguments as to whether imagery is pictorial (Cole et al., 2022; Pylyshyn, 2002), there is now a wealth of research indicating that imagery is multimodal (Lacey & Lawson, 2013b), encompassing any of the sensory modalities: visual, auditory, tactile, gustatory, olfactory, proprioceptive, vestibular, thermoceptive, or visceroceptive. Research has focused much on visual (Pearson, 2019; Takahashi et al., 2023), auditory (Hubbard, 2010; Zatorre et al., 1996), and motor imagery (Toth et al., 2020). Given that imagery can be understood as an internal perceptual-like “copy” (=imago) of the external world, it also conceivably differs based on the perceptual qualities found in the external world, such as pitch, rhythm, darkness, brightness, clarity, and vividness (Hubbard, 2010, 2018).

### Mental imagery overlaps with perception

Mental imagery has been described as a shadow of perception (Kosslyn et al., 2010), because of shared perceptual processes and properties. Evidence from numerous studies indicates that during imagery tasks, perceptual systems are active, for instance for visual imagery the primary visual cortex (Pearson, 2019), but also the hippocampus (Bird et al., 2010), and frontal areas (Yomogida et al., 2004). Further overlaps exist between memory and imagery (with perception, Addis, 2020). In any case, the experience of conscious mental imagery appears to rely, in part, on processes used in perception.

### Large individual differences in conscious experience of mental imagery exist

Importantly, it is likely that individual differences exist in how explicit, or conscious, imagery is (Brogaard & Gatzia, 2017). In what has been similarly termed “mind blindness” or aphantasia (Zeman et al., 2015), around four percent report experiencing no (conscious) mental imagery. Although much cognitive processing can be implicit, the idea of unconscious or implicit mental imagery is particularly analogous to the phenomenon of blindsight, whereby people can use their visual system to interact with the environment without conscious visual experience (Cowey, 2010). Consistent with the idea that people with aphantasia might still experience implicit imagery, there is very little evidence that people with aphantasia suffer cognitive deficits (Cavedon-Taylor, 2022; Dawes et al., 2020). Finally, even “normal” imagers do not create mental images all the time (Isaac & Marks, 1994).

### Approaches to measuring mental imagery

A subject of much research and debate has been the measurement of mental imagery (Pylyshyn, 2002). If mental imagery is multimodal, perceptual-like, and shows individual differences in conscious experience, then it would be advantageous to have a measure of mental imagery that, in turn, can measure perceptual-like imagery across multiple modalities, even for those where less conscious imagery is experienced. Before presenting such a measure, a review of other measures is firstly provided, based on a meta-analysis that identified 65 measures of mental imagery (Suica et al., 2022). Briefly, previous measures can be grouped into those that are self-report and those that are performance related.

### Self-report measures of mental imagery

The vast number of the 65 measures identified are self-report scales and questionnaires, mostly for assessing motor imagery (Suica et al., 2022). A number of self-report general measures of mental imagery have been developed, three of which are in common usage. The first is a self-report 32-item scale called the Vividness of Visual Imagery Questionnaire (VVIQ) (Isaac & Marks, 1994). One disadvantage with the VVIQ is that it measures only visual imagery, ignoring the other seven or eight sensory modalities. The Plymouth Sensory Imagery Questionnaire (PSIQ) measures self-reported imagery across seven modalities, namely the five classic senses, plus kinesthetic and emotional imagery (Andrade et al., 2014). Also commonly used, the Spontaneous Use of Imagery Scale (SUIS) measures the extent to which participants report experiencing images spontaneously (Görgen et al., 2016).

Self-report scales are problematic for a number of reasons (see Dahm, 2020). First, they tend to capture explicit mental imagery (e.g., “how well can you imagine a sunrise”), which makes it difficult to capture imagery in participants where this is more implicit. Second, it would seem reasonable to assume that self-report scales cannot be used to reliably measure individual differences, because doing so would require the respondent to be able to rate not only their own imagery, but that of other people. This position is controversial, as it may be taken to call into question the use of questionnaires in psychology generally, however, it is here intended as a limitation, not a fatal flaw, which necessitates use of other objective measures alongside. Third, the scales rely on a limited number of scenarios (usually between 5 and 10), hence representing a relatively small behavioral sample.

### Mental imagery performance tasks

A smaller group of measures have been identified as assessing mental imagery more objectively. By objective, allowing comparable performance across participants is meant, on a task that can be reasonably observed from a test administrator to have been solved correctly or not.

#### Motor movement tasks

Several tasks have been developed that attempt to objectify motor imagery. The German Test of the Controllability of Motor Imagery in Adults (Schott, 2013) requires participants to listen to six movement instructions, imagine them, and then at the end to execute the movement. The movement is then rated as to whether it matches with the instructions. Similarly, the Test of Ability in Movement Imagery (Madan & Singhal, 2013) asks participants to imagine a motor movement presented as a verbal instruction and then to select a picture from a range of options that matches with the described movement. Both tasks contain several difficulties regarding their measurement of actual mental imagery. First, it is conceivable that the participants execute the final movements based on memorized verbal instructions, hence the task could be solved propositionally. Second, the task would appear to rely on complex verbal processing to translate the instructions into images or movements. Third, because of having to store six instructions or images in memory, the task is likely dependent on working memory, which accordingly constitutes a potential subject-variable confound. Fourth, and exclusive to the Test of Ability in Movement Imagery, participants then have to match an image of their new position onto a photographically present image of someone else in that position. This likely introduces a further skill demand to the requirements of working memory and verbal transformation. In short, for both tasks, the causal chain and underlying cognitive processes from instruction to completion appears too long to be sure what exactly is being measured.

#### Hand laterality tasks

A second set of tasks concern left/right positioning. In hand laterality tasks, hands are presented in a certain position and one has to respond by saying whether the depicted hand is a left hand or right hand. Similarly, left/right judgement tasks require participants to determine whether a body part is on the right of left side (Pelletier et al., 2018). Both of these tasks would appear to capture one small aspect of imagery and rely on participants’ having equal mastery of left and right orientations.

#### Cube cutting task

The Cube Cutting Task (Lorenz & Neisser, 1985) requires participants to follow a set of complicated verbal instructions to imagine a 3 × 3 × 3 cube and then perform certain cuts into the cube and mentally count the number of smaller cubes in the obtained geometric forms. Again, this task requires complex conversion of verbal/written stimuli into visual forms, which might be confounded by individual differences in the ability to do so. Similar tasks involve participants to mentally jog through a series of images and count features (e.g., how many windows does your house have? Or, how many lower-case letters of the alphabet contain no straight lines?).

#### Chronometric tasks

Chronometric tasks are behavioral measures that attempt to capture individual differences in imagery, usually through measuring processing speeds from imagery processing. Some tasks use priming for imagery in one sensory modality to test stimulus processing in another modality. However, it is not clear whether imagery causes the processing advantages or whether it is a cross-modality attentional cost (Hubbard, 2018).

#### Mental rotation tasks (MRT)

MRT have a rich history in psychology and have the clear advantage of being objective, in the sense that performance can be timed precisely and evaluated accurately (Shepard & Metzler, 1971). Although a number of strategies are possible, it appears that solving MRT for most participants involves encoding, searching, and comparing the reference and target stimuli (Xue et al., 2017). Further, it could be reasonably expected that once perceptual encoding of the stimuli is adequate, a mental rotation of the one object onto the other ensues, to test the hypothesis that both stimuli match. This last step is thought to involve the mental imagery skill of manipulation and inspection; accordingly, mental rotation tasks are often experienced as being quite cognitively taxing involving cognitive processes such as working memory (Pardo-Vazquez & Fernandez-Rey, 2012). Further, it could be argued that the MRT is a very distal type of imagery to that understood here for two reasons. First, mental rotation is only minimally multimodal, relying heavily on visual information. However, this may depend on the stimulus as a variant of the MRT involving the rotation of human bodies appears to relate to action processing (Dahm, 2020; Steggemann et al., 2011). Second, the experience of rotating an object is quite different from imagining a perceptual stimulus, hence might have limited ecological validity as a measure of mental imagery – particularly as it is generally accepted that only one of the steps in performing mental rotation tasks is thought to require imagery (Xue et al., 2017).

#### Mental comparison tasks (MCT)

A task that has long been used informally to assess mental imagery skill is the MCT. In this task, participants are asked to make a mental comparison between two objects, in the absence of those objects and on a sensory property relating to both that is generally held to be objectively true (Moyer, 1973). Variants have featured in research on auditory imagery (Hubbard, 2010; Zatorre et al., 1996) and the MCT can seemingly be administered to both adults and children (Suggate & Lenhard, 2022; Suggate & Martzog, 2020).

Recently, a version of the MCT was developed to capture individual differences in mental imagery as an implicit/explicit perceptual-like process, across different sensory modalities (Martzog & Suggate, 2019). The MCT was computerized and standardized to provide reliable measures of participants’ reaction times and response accuracy (Suggate & Lenhard, 2022; Suggate & Martzog, 2020). Specifically, two comparison items and a sensory property are provided, in the format of ‘*what is* [sensory property, e.g., “*softer*”], [object 1 “*a pine needle*”] or [“*a blade of grass*”]?’ The sensory property can be selected from any sensory modality for which there are enough appropriate lexemes. The comparison items have generally been selected to be unambiguous, defined as approximately 80% of respondents agree (which is shinier/louder etc.). In selecting items, and also by selecting a large number of items, the goal is to ensure that participants are unlikely to be able to solve the task propositionally (Zatorre et al., 1996), that is by having learnt explicit facts about the comparison (e.g., that violins are not particularly shiny). Moreover, the task appears time economical (taking about 5 s per item) and can assess imagery across a range of sensory modalities (e.g., visual, tactile, auditory).

Although promising, work has only recently been conducted with the MCT as a performance measure. The MCT has shown excellent internal consistency (Suggate & Lenhard, 2022), with a short version of the task correlating moderately over 1 year in kindergarten age children (*r* = .27 for response latency and *r* = .58 for accuracy, Suggate & Martzog, 2020). The MCT has shown predictive validity, correlating with adult reading performance (Suggate & Lenhard, 2022) and children’s fine motor skills (Martzog & Suggate, 2019), indicating that the MCT relates to sensorimotor simulation and higher-order cognitive processes (i.e., reading comprehension). Further, findings indicate that MCT performance can be suppressed after children’s (Suggate & Martzog, 2020; Suggate & Martzog, 2022) and adults’ (Suggate, 2023) media usage, perhaps because screen-media may reduce active mental image generation (Valkenburg & van der Voort, 1994). Although this work shows promise regarding the construct validity of the MCT, the task needs further development.

### Current studies

In the current studies, the MCT is examined as a potential objective measure of mental imagery. Mental imagery in turn is understood as being multimodal, varyingly conscious, and perceptual-like. The potential advantages of the MCT over commonly used self-report measures are firstly that this allows objective measurement of individual differences, going beyond self-report. Second, by including a larger number of items than typical, a sufficient sample of mental images can be taken making it less likely that overall performance can be explained by confounds in the individual items. Third, different sensory aspects of mental imagery can be tested, pertaining to various sensory modalities. Fourth, given the comparative simplicity of the task – compared to, for example, the Cube Cutting Test – fewer confounding mental processes might be expected, such as executive functioning and working memory. Fifth, although not directly tapping important constructs related to imagery quality, such as vividness and detail possible in self-report questionnaire (e.g., “imagine a setting sun”), the MCT would still appear to capture a key dimension of mental imagery focused on the perceptual symbols comprising more complex images (Brogaard & Gatzia, 2017). However, there are still key questions as to the performance of the MCT and to what extent this measures mental imagery. Accordingly, across three studies, the following research aims were pursued, namely to:

1. establish variance and mean, internal consistency, and the speed–accuracy correlation of the MCT
2. test for correlations with self-report imagery scales as a means to validate both, also with regards to conscious imagery
3. examine whether MCT performance is affected by semantic features
4. investigate MCT performance across different sensory modalities (visual, tactile, auditory)
5. determine whether processing speed explains mental imagery performance

## Study 1

In Study 1, the MCT is administered to adults and validated against two self-report measures of mental imagery, namely the SUIS and the PSIQ. In this first study, the MCT was selected to assess imagery from visual, visual-tactile, and haptic/tactile modalities to provide a range of experiences not solely in the visual modality. The SUIS was selected because it purports to measure the spontaneous appearance of mental imagery, which might relate to image generation processes in the MCT. The PSIQ was selected because it attempts to measure imagery as a multimodal construct, as does the MCT. Accordingly, it was hypothesized that the MCT would show good internal consistency and correlate with both the PSIQ and the SUIS.

Additionally, it was assumed that there would be a negative correlation between response latency and accuracy, whereby faster responses were also generally more accurate. Although the phenomenon of a speed–accuracy trade-off exists (Salthouse, 1979), this is more likely when participants are asked to respond with a focus on speed, not accuracy (Standage et al., 2016). Given that the instructions for the MCT required participants to respond accurately and quickly, a negative correlation was expected. Further, assuming that imagery is perception-like, such a correlation would also provide insight into whether faster image generation and inspection relates to greater imagery accuracy.

### Methods

#### Participants

Participants were 96 university teacher education students attending a first-year lecture in Germany and aged 21.35 years (*SD* = 3.91). Participation was voluntary but recommended as an opportunity to take part in research and the results were discussed with the students at the end of the course. Due to a programming omission, gender was not collected in this sample. Participation was voluntary but encouraged to provide students experience of experimental research as part of course participation.

#### Measures

First demographic questions were administered (i.e., age, gender, tertiary degree, country of birth) and all measures were conducted in German.

##### Plymouth Sensory Imagery Questionnaire (PSIQ)

The PSIQ was selected as a self-report measure tapping mental images in seven sensory domains: vision, hearing, touch, taste, smell, body, and emotional imagery (Andrade et al., 2014). Each sensory modality has five items answered on an 11-point Likert scale (from “no mental image” to “clear and alive, like in real life”). Scores were combined to create a single imagery score out of the sum of all items and the internal consistency was high, α_cr_ = .91.

##### Spontaneous Use of Imagery Scale (SUIS)

The SUIS was used as a second self-report measure of mental imagery (Görgen et al., 2016) because it was reasoned that spontaneously experiencing mental images might relate to MCT performance. The SUIS is a 12-item scale measuring participants’ immediate and everyday experience of imagery. In the German adaptation, six items were added (Görgen et al., 2016). Participants respond to statements such as “When I listen to the news, real-life images appear in my mind” on a five-point Likert scale (1 = “never” to 5 = “always”). The scale has been shown to be unidimensional (Görgen et al., 2016) and correlates with the PSIQ (Andrade et al., 2014). In the current study the internal consistency was high, α_cr_ = .80.

##### Imagery comparisons task (MCT)

An MCT was used to measure participants’ mental imagery (Moyer, 1973; Paivio, 1975, for further development see: Martzog & Suggate, 2019). As outlined above, for each item participants were asked to imagine two words and make a sensory judgment (visual and/or tactile) based on a property of the stimuli’s corresponding mental images. For instance, “what is shinier, trumpet or violin?” Thus, the question “what is” was played at the beginning of the trial (0 s), followed by the adjective (e.g., “pointier”, at 1 s), then the first target imagery item (“a nail”, at 2 s), “or” (at 4 s), and then the second target imagery item (“a needle”, at 5 s). Participants were instructed to respond as quickly and accurately as possible by pressing the “f” key for the first target item and the “j” key for the second. The presentation of each target imagery item was accompanied with a marker (i.e., a small square and the corresponding key press) on the left and right sides of the screen, to serve as a guide as to which key press was paired with which stimulus.

Response accuracy and latency were both recorded and in total there were 44 MCT items across the visual (i.e., brighter, shinier), tactile (i.e., scratchier, softer, sharper), and visual-tactile (i.e., larger, pointier) modalities. Response latency was conceptualized as being the key dependent variable, particularly in adult samples, given that it was expected that participants would be able to answer close to all items correctly. Item order was randomized for each individual participant. Internal consistency for (winsorized) response latency was α_cr_ = .96, *r*_SB_ = .81, and for response accuracy was α_cr_ = .85, *r*_SB_ = .88.

##### Procedure

The study was an online correlational study programmed in PsychoPy 2020.1.3 and run in Pavlovia (Peirce et al., 2019), which has demonstrated timing accuracy across a range of browsers of less than 3.5 ms (Bridges et al., 2020). Participants first answered demographic questions and then performed the PSIQ, SUIS, followed by questions on reading habits not reported here, and the MCT. In total, the study lasted approximately 20 min. The research was conducted in accordance with university guidelines, the Helsinki Declaration, and APA ethics standards. Because Study 1 was exploratory, the significance level of *p* = .05 was selected.

### Results and discussion for Study 1

MCT response latencies were capped at 10 s (*n* = 11) in a Winsorization procedure to avoid implausibly long times biasing findings and to remove fast responses at a low accuracy (below 75%). This resulted in winsorizing 4.82% of data at .40 s and .24% of data at 10 s. Four participants were then excluded because their response accuracy on the MCT was below 75%, leaving 92 participants. All individual items were responded to at an accuracy rate of greater than 75% (two items at 79%, 14 items 81% to 90%, and 28 items ≥ 90%), indicating that there was little ambiguity across participants as to the correct response. Descriptive statistics for the MCT, PSIQ, and SUIS measures were calculated and appear in Table 1, along with correlations between these measures.

**Table 1:** Descriptive statistics and correlations between the aggregated imagery measures in Study 1

		*M*	*SD*	*N*	Min.	Max	1	2	3	4
1	MCT (rt)	1.21	.57	92	.67	4.76	-	– .12	– .12	.17
2	MCT (acc)	91.72	4.58	92	79.50	100.00		-	– .13	– .06
3	PSIQ	253.75	39.03	92	153	337			-	.37*
4	SUIS	61.28	10.58	92	31	83				-

MCT = mental comparison task, rt = response time/latency, acc = accuracy expressed as a proportion between 0 and 1, PSIQ = Plymouth Sensory Imagery Questionnaire, SUIS = Spontaneous Use of Imagery Scale

^*^= p < .05

As reported in the measure section, the MCT returned high levels of internal consistencies (> .90) and as reported in Table 1, participants’ response latencies were 1.21 s (CI 1.09–1.33). It may be useful to compare these response latencies with other tasks to gain an idea whether image generation and inspection might reasonably have occurred, as hypothesized. Go/no-go tasks involving a similar sample to here requiring participants to judge whether a high-frequency word versus a non-word is a real word yield responses of around 600 ms (Perea et al., 2002). Slightly slower responses have been obtained in tasks involving semantic decisions about lexical stimuli (e.g., is it an animal) of around 700 ms (Siakaluk et al., 2003). A lexical decision task requiring a judgment about a category (e.g., is it an animal) is not too dissimilar to the MCT which involves a perceptual judgment about an image (e.g., is it pointier). Accordingly, the leap in response latencies from 700 ms to approximately 1200 ms found here suggests that it is plausible that image inspection and comparison is occurring in this time. However, this is a rough-hewn and not a like-for-like comparison, contrasting semantic processing with perceptual processing.

Turning to the hypothesis that MCT response latency and accuracy would be negatively correlated, Table 1 shows a non-significant correlation. This finding is surprising and may well be due to the small number of observations in the correlational analysis in Table 1 (*n* = 92) taken at an aggregate level (i.e., mean response latency per participant across all trials). Accordingly, a post hoc second correlation was conducted at the item level, whereby individual response latencies to each item were correlated with response accuracy. This yielded a significant small negative correlation, *r*(4047) = – .11, *p* < .001, in support of the hypothesis. On the item level, this indicates that easier items have shorter response latencies and higher response accuracies than more difficult items, which was also confirmed with *t* tests.

Findings indicated that the PSIQ and the SUIS were significantly correlated, but their correlations with the MCT were not. Accordingly, the hypothesis that the MCT would correlate with both self-report measures was not supported. Conceptually, three possibilities exist for this null finding. First, the current sample size might have been too small to detect differences. Sond, it might be that self-report responses are not directly comparable between participants, because participants fundamentally cannot rate how their imagery compares to somebody else’s, such that these provide an inaccurate estimate of participants’ between-subject imagery abilities. Thus, it might still be assumed that the MCT does measure mental imagery abilities, whereas the self-report scales instead mostly measure potentially fallible within-subject self-rated imagery abilities. In contrast, the positive correlation in responses between the SUIS and the PSIQ, supports the idea that self-report scales (reliably) capture within-subject differences. However, the correlation is small to moderate using Cohen’s standards and lower than that expected for two measures tapping the same construct with a similar method. Third, it is possible that the MCT and the self-report measures capture different aspects of mental imagery. Self-report scales may assess explicit mental imagery experience or vividness whereas the MCT captures the ability to construct and compare sensorimotor simulations of external events. These possibilities will be further explored in Study 2.

## Study 2

Study 1 found that the MCT was reliable (in terms of internal consistency) and that more accurate responses were also faster. Further, the MCT resulted in response latencies consistent with the formulation and inspection of mental images; however, this performance did not relate to self-report scales. One reason for this lack of correlation could be due to the sample size. Accordingly, one aim of Study 2 was to replicate Study 1 using a larger sample size in terms of both participants and the number of MCT items. The latter was increased to provide a more stable estimate of imagery abilities and, with the advantage of avoiding potential response fatigue, administered in smaller blocks of seven items.

Additionally, given that the MCT items are presented (orally) as words, it is possible that response latencies due to imagery are confounded by lexical features of the individual items, as has been found in much research (Pexman et al., 2019). Obvious candidates are the length of the words (in syllables) and the frequency with which the word occurs in language, as both features relate to lexical processing times (Suggate & Stoeger, 2017). Accordingly, MCT performance was modeled as a function of these item-level lexical covariates.

Third, mental imagery is hypothesized to be a multi-modal simulation of perceptual-like events. To test this idea, MCT items were selected that pertained to three sensory modalities, namely the visual, auditory, and tactile modalities. Thereby, Study 2 can test whether responses differ as a function of modality. For instance, tactile information is processed more slowly than visual, which in turn is processed more slowly than auditory information (Barnett-Cowan & Harris, 2009). Accordingly, if imagery processing is supported by the sensory modalities analogous to sensory processing, it might be expected that tactile imagery is also slower than visual and auditory imagery. A further advantage is that the PSIQ also includes (seven) different modalities, thus a more fine-grained validation of the MCT is possible, by correlating MCT visual, visual-tactile, and tactile responses with corresponding PSIQ modalities.

It was hypothesized that both number of syllables and word frequency would relate to MCT performance and that tactile imagery would be processed more slowly than auditory or visual imagery. Second, it was expected that the SUIS and PSIQ would show small but significant correlations with the MCT given the larger sample size. Third, taking advantage of the PSIQ measuring self-report mental imagery across seven modalities, it was expected that self-reported visual, auditory, and tactile imagery would correlate more strongly with the corresponding modality on the MCT than with other modalities.

### Methods

#### Participants

The participants (*n =* 345) were again students attending a first-year education lecture, 97.09% were born in Germany, 29.4% identified themselves as male and 70.6% as female, 80.50% were studying in their first semester, and were aged 19.95 years (*SD* = 2.34).

#### Measures and procedure

As with Study 1, measures included the MCT, SUIS, and PSIQ. The MCT was administered with four key differences. First, the number of items was expanded to include 126 items (seven items per block, across six blocks, over three modalities), which are presented in the Appendix. Second, three modalities were included, namely the visual (brighter), auditory (louder), and tactile (softer/scratchier). Third, a practice set of 13 items was included at the beginning of the experiment, whose data were not analyzed here. Fourth, as part of another study, the items were administered in blocks of seven items, followed by either the viewing of a 1-min film clip or reading a text for 1 min. A single-factor ANOVA with LSD post hoc tests revealed that the first item in each block of seven MCT trials was responded to more slowly, *F*(6, 28973) = 29.24, *p* < .001, but not less accurately, *F*(6, 28973) = .25, *p* = .96. Because presentation of the items was randomized within each block, this did not systematically affect any single items. For multilevel models, the decision was made to include a dummy variable for the first items so as to maximize statistical power, yet provide a baseline estimate for a typical MCT item by partialing out this first-item-in-block effect. Because a key purpose of Study 2 was exploring links between the PSIQ and the MCT, alpha was set at *p* =.05.

To account for a potential confounding influence of lexical factors on the MCT, the number of syllables was counted for each word in the MCT. Further, the frequency with which the words occur in the German language was estimated using lexica corpora (Quasthoff & Richter, 2005). Higher numbers represent less frequent words, derived from the log base 2 of the absolute frequency rank of the given word compared to the most frequent word (e.g., in English, “the” is the most frequent word, which has a class of 0). Thus, a word with a class of 8, or 15, appears twice as often as that with a class 9, or 16, respectively. To obtain an item level estimate, the word length and frequency mean was taken across both stimuli in each mental comparison. The internal consistency and split-half reliability of the MCT was excellent for both accuracy, α_cr_ = .86, *r*_SB_ = .84, and speed, α_cr_ = .99, *r*_SB_ = .84.

### Results and discussion

Extreme outliers were treated by winsorizing at 10 s and at .35 s because responses faster than .35 s were less than 75% accurate. This resulted in excluding .39% of data at the slower and .57% of data at the faster end. Further, six items, four in the tactile and one each in the visual and auditory modalities, had lower response accuracies (ranging from 48% to 72%) and hence were dropped. Descriptive statistics for the MCT are presented in Table 2, including a breakdown by MCT modality. As can be seen in Table 2, responses appeared fastest for the visual, followed by the auditory and tactile modalities, *d* = .20 for visual vs. tactile, *d* = .15 for visual versus auditory. This pattern was confirmed using a multivariate ANOVA, *F*(2, 41397) = 145.65, *p* < .001 for response latency, *F*(2, 41397) = 15.04, *p* < .001, and the resulting post hoc LSDs were all significant, *p* < .001. For response accuracy, visual and auditory did not differ, *p* = .21, but were both more accurate than tactile response latencies, *p* < .001.

**Table 2:** Descriptives statistics for the mental imagery tasks in Study 2

Modality	Unit	*M*	*SD*	*n*	Min	Max	Skew	Kurt
MCT all	acc	.95	.22	41400	0	1	– 4.01	14.66
MCT all	s	1.38	.90	41400	.35	10	4.68	33.14
Visual	acc	.95	.21	14145	.00	1.00	17.05	– 4.36
Visual	s	1.28	.88	14145	.35	10.00	36.15	4.93
Tactile	acc	.94	.24	14145	.00	1.00	11.88	– 3.72
Tactile	s	1.46	.92	14145	.35	10.00	30.54	4.51
Auditory	acc	.95	.22	14145	.00	1.00	15.59	– 4.19
Auditory	s	1.41	.88	14145	.35	10.00	34.24	4.75
SUIS	acc	3.54	0.57	345	1.11	5.00	– .52	.90
PSIQ	acc	6.89	1.09	345	1.66	9.00	– .79	1.61
Visual	acc	7.61	1.18	345	1.80	9.00	– 1.14	1.93
Auditory	acc	7.20	1.39	345	0.80	9.00	– 1.05	1.36
Olfactory	acc	6.44	1.54	345	1.00	9.00	– .61	.18
Gustatory	acc	6.59	1.56	345	1.00	9.00	– .61	.13
Tactile	acc	7.14	1.40	345	1.00	9.00	– 1.14	1.70
Kinesthetic	acc	6.93	1.36	345	.80	9.00	– .83	1.32
Emotional	acc	6.29	1.63	345	.00	9.00	– .84	1.31

MCT = mental comparison task, PSIQ = Plymouth Sensory Imagery Questionnaire, SUIS = Spontaneous Use of Imagery Scale, acc = accuracy expressed as a proportion between 0 and 1

Correlation coefficients between the MCT and self-report measures were conducted using subject-level mean scores across the corresponding items and these appear in Table 3. As Table 3 shows, the MCT modalities correlated highly strongly with each other, and the PSIQ modalities also correlated strongly with each other, and to a lesser extent with the SUIS. There were small but statistically significant correlations between some of the MCT scales and the PSIQ subscales.

**Table 3:** Pearson correlation coefficients for the self-report imagery scales in Study 2

		1	2	3	4	5	6	7	8	9	10	11	12	13	14
1	MCT mean (rt)	-	– .28^*^	.96^*^	.96^*^	.96^*^	.08	.03	.00	.12^*^	.11^*^	.00	.06	.09	– .02
2	MCT mean (acc)		-	– .21^*^	– .30^*^	– .30^*^	.16^*^	.26^*^	.28^*^	.04	.00	.16^*^	.16^*^	.02	.09
3	MCT tactile (rt)			-	.87^*^	.88^*^	.09	.05	.04	.12^*^	.09	.01	.06	.08	– .02
4	MCT visual (rt)				-	.88^*^	.07	.02	– .02	.13^*^	.09	.00	.07	.08	– .03
5	MCT auditory (rt)					-	.07	.00	– .02	.11^*^	.12^*^	– .01	.06	.08	– .02
6	PSIQ mean						-	.70^*^	.76^*^	.78^*^	.75^*^	.85^*^	.83^*^	.67^*^	.34^*^
7	PSIQ vision							-	.63^*^	.41^*^	.44^*^	.59^*^	.51^*^	.30^*^	.23^*^
8	PSIQ sound								-	.46^*^	.47^*^	.62^*^	.56^*^	.37^*^	.26^*^
9	PSIQ smell									-	.59^*^	.59^*^	.61^*^	.46^*^	.31^*^
10	PSIQ taste										-	.59^*^	.53^*^	.33^*^	.19^*^
11	PSIQ touch											-	.70^*^	.49^*^	.27^*^
12	PSIQ kinesthetic												-	.50^*^	.28^*^
13	PSIQ emotion													-	.25^*^
14	SUIS mean														-

MCT = mental comparison task, rt = response time/latency, acc = accuracy expressed as a proportion between 0 and 1, PSIQ = Plymouth Sensory Imagery Questionnaire, SUIS = Spontaneous Use of Imagery Scale

^*^ *= p* < .05

Next multilevel linear models were conducted to test (a) the statistical relation between MCT response accuracy and latency, (b) examine whether the lexical features of word length and frequency affected MCT performance, and (c) test the relation between the self-report imagery scales and the MCT. Multilevel linear models have the advantage of modeling item-level and participant-level effects in nested data structures, which are present here (Raudenbush & Bryk, 2002). Models were run with maximum likelihood estimation and included random intercepts at the participant and item levels. A dummy variable for the first MCT trial in each block was included to partial out the influences of slower first item response latencies. A model was first run without the self-report imagery measures to test the contribution of word length and frequency and MCT response accuracy was included as a predictor to test relations with response latency. Both models are presented in Table 4.

**Table 4:** Multilevel linear models predicting mental comparison task (MCT) response latencies in Study 2

	MCT response latency			MCT response latency		
Predictors	Estimates	CI	*p*	Estimates	CI	*p*
(Intercept)	1.55	1.41 – 1.69	<.001	1.40	1.03 – 1.77	<.001
MCT response accuracy	– .42	– .45 to – .38	<.001	– .42	– .45 to – .38	<.001
MCT first item centered	.18	.16 – .20	<.001	.18	.16 – .20	<.001
Mean syllables	.02	– .02 – .06	.242	.02	– .02 – .06	.242
Mean frequency	.02	.01 – .04	.011	.02	.01 – .04	.011
SUIS mean				– .04	– .13 – .04	.332
PSIQ mean				.04	– .00 – .09	.053
Random effects						
σ^2^	.57			.57		
τ_00_	.18 _subject_			.18 _subject_		
	.03 _item_			.03 _item_		
ICC	.26			.26		
N	345 _subject_			345 _subject_		
	120 _item_			120 _item_		
Observations	41400			41400		
Marginal R^2^ / Conditional R^2^	.00 / .28			.02 / .28		

MCT = mental comparison task, PSIQ = Plymouth Sensory Imagery Questionnaire, SUIS = Spontaneous Use of Imagery Scale

The models in Table 4 show that response accuracy was significantly and negatively related to response latency, consistent with Study 1 and the hypothesis. Word frequency predicted response latencies, but word length did not. Finally, consistent with Study 1, but not consistent with the hypotheses, none of the self-report questionnaires significantly predicted MCT response latencies. However, the coefficient with PSIQ was larger than that for word frequency, yet not statistically significant, probably due to the large variance in the PSIQ. Accordingly, further analyses looked at the PSIQ in more detail.

Thus, three models were run in which the MCT was teased apart into responses to items pertaining to each of the three modalities (i.e., visual, auditory, tactile). The PSIQ was broken down into scales representing each of the seven imagery modalities that it reports to measure and these were entered as predictors. Word frequency and response accuracy were retained as a control variable because this was a significant predictor in Table 4. The models are presented in Table 5. Contrary to hypotheses, there was little systematic evidence that PSIQ ratings for the visual, auditory, and tactile modalities related to the corresponding modalities in the MCT.

**Table 5:** Multilevel linear models predicting mental comparison task (MCT) response latencies as a function of Plymouth Sensory Imagery Questionnaire (PSIQ) modality in Study 2

	MCT visual			MCT auditory			MCT tactile		
*Predictors*	Estimates	CI	*p*	Estimates	CI	*p*	Estimates	CI	*P*
(Intercept)	1.33	.92 – 1.74	<.001	1.43	1.05 – 1.82	<.001	1.34	.96 – 1.73	<.001
MCT response accuracy	– .45	– .51 to – .39	<.001	– .40	– .47 to – .34	<.001	– .41	– .47 to – .35	<.001
Word frequency	.02	– .01 – .05	.204	.02	– .00 – .04	.080	.02	.00 – .04	.014
MCT first item dummy	.23	.19 – .26	<.001	.16	.12 – .19	<.001	.16	.12 – .19	<.001
PSIQ vision	.02	– .03 – .07	.475	.01	– .04 – .06	.726	.03	– .03 – .08	.379
PSIQ sound	– .03	– .07 – .02	.239	– .02	– .07 – .02	.344	– .00	– .05 – .05	.956
PSIQ smell	.04	– .00 – .08	.076	.03	– .01 – .07	.206	.03	– .01 – .08	.133
PSIQ taste	.02	– .02 – .06	.270	.04	.00 – .08	.030	.02	– .02 – .06	.328
PSIQ touch	– .05	– .10 – .00	.065	– .06	– .12 to – .01	.019	– .06	– .12 – .00	.051
PSIQ kinesthetic	.02	– .03 – .07	.407	.02	– .03 – .07	.508	.01	– .05 – .06	.833
PSIQ emotional	.02	– .02 – .05	.379	.02	– .01 – .06	.171	.02	– .02 – .06	.300
Random effects									
σ^2^	.54			.56			.59		
τ_00_	.17 _subject_			.17 _subject_			.20 _subject_		
	.03 _item_			.02 _item_			.02 _item_		
ICC	.27			.25			.27		
N	345 _subject_			345 _subject_			345 _subject_		
	41 _item_			41 _item_			38 _item_		
Observations	14145			14145			13110		
Marginal R^2^ / Conditional R^2^	.03/.29			.03/.28			.03/.29		

MCT = mental comparison task, PSIQ = Plymouth Sensory Imagery Questionnaire, SUIS = Spontaneous Use of Imagery Scale

Part of the reason for the lack of significant correlations between the PSIQ and the MCT could be due to the fact that one measures response latency and the other purported vividness of imagery, which might represent different constructs (Lacey & Lawson, 2013a, 2013b). Accordingly, a new set of models were run. These models were similar to those in Table 5 but predicted MCT response accuracy instead. The models presented in Table 6 generally find that the PSIQ relates more strongly to the MCT accuracy scores. Concerning the specific hypotheses, PSIQ visual scores related to MCT visual accuracy, as did PSIQ sound to MCT auditory. Accordingly, using response accuracy for the MCT, limited support was found for the hypothesis that MCT modalities would relate to PSIQ modalities.

**Table 6:** Multilevel linear model predicting mental comparison task (MCT) response accuracy as a function of Plymouth Sensory Imagery Questionnaire (PSIQ) modality in Study 2

	MCT visual (acc)			MCT auditory (acc)			MCT tactile (acc)		
Predictors	Estimates	CI	*p*	Estimates	CI	*p*	Estimates	CI	*P*
(Intercept)	.88	.81 to .96	<.001	.82	.75 to .89	<.001	.83	.75 to .90	<.001
Word frequency	– .01	– .01 to .00	.140	.00	– .00 to .01	.792	.00	– .00 to .01	.424
MCT first item centered	– .00	– .01 to .01	.707	.00	– .01 to .01	.869	– .01	– .02 to – .00	.033
PSIQ vision	.01	.00 to .02	.037	.01	.00 to .02	.008	.01	– .00 to .02	.066
PSIQ sound	.01	.01 to .02	<.001	.01	.01 to .02	<.001	.01	.00 to .02	.011
PSIQ smell	– .00	– .01 to .01	.763	– .00	– .01 to .01	.551	– .00	– .01 to .00	.166
PSIQ taste	– .01	– .02 to – .00	.001	– .01	– .02 to – .00	.008	– .01	– .01 to .00	.063
PSIQ touch	.01	– .00 to .01	.119	– .00	– .01 to .01	.509	.00	– .01 to .01	.715
PSIQ kinesthetic	.00	– .01 to .01	.786	.00	– .00 to .01	.354	.01	– .00 to .02	.078
PSIQ emotional	– .01	– .01 to – .00	.035	– .00	– .01 to .00	.517	– .01	– .01 to .00	.072
Random effects									
σ^2^	.04			.04			.05		
τ_00_	.00 _subject_			.00 _subject_			.00 _subject_		
	.00 _item_			.00 _item_			.00 _item_		
ICC	.12			.12			.09		
N	345 _subject_			345 _subject_			345 _subject_		
	41 _item_			41 _item_			38 _item_		
Observations	14145			14145			13110		
Marginal R^2^ / Conditional R^2^	.02/.13			.02/.13			.01/.10		

MCT = mental comparison task, PSIQ = Plymouth Sensory Imagery Questionnaire

Accordingly, Study 2 builds on Study 1 in several key ways. First, it confirms that MCT response latency and accuracy were significantly negatively correlated. Second, controlling for word frequency and length did not fundamentally alter relations found with the MCT. Third, there was some evidence that self-report imagery measures correlated with MCT accuracy. Fourth, it found that responses were slower and less accurate for tactile items and fastest for visual items.

## Study 3

The MCT involves making decisions about sensory properties of two imagined stimuli, as quickly as possible. Accordingly, it is possible that the MCT measures processing speed, either in addition to or instead of mental imagery. Study 3 tested this contingency by employing a task with an identical construction to the MCT that, however, instead of images, two sounds at different volumes were compared. Specifically, following the question “what is louder”, two short auditory beeps are presented and response latencies and accuracies are recorded. By using the exact same task format and timings as the MCT, a close match for processing speed is provided, with one key difference between the two tasks being whether stimuli or images are compared. Also, by using a sound comparison task the stimuli are presented in a different modality to the visual and tactile imagery modalities invoked in the MCT. This was reasoned to be necessary to meet the conditions of being a processing speed control, without explaining away underlying visual/tactile imagery processes.

Further, in addition to including the PSIQ, the MCT modalities were correlated with each other and with processing speed. It was hypothesized that the MCT tasks would correlate more strongly with each other than with processing speed, and that the MCT would correlate with each other after controlling for processing speed. Finally, in line with Studies 1 and 2, it was not expected that the general absence of relations between the MCT and the PSIQ would change after controlling for processing speed.

### Methods

#### Participants

Participants were originally 465 university students who were invited to participate as part of an introductory education lecture. The students were aged between 17 and 38 years (*M* = 20, *SD* = 2.5), 99% were born in Germany, and an estimated 79.2% (*n* = 366) were female. Additionally, 99% had attained university entrance qualifications and 21% had already received a first degree in higher education. Participants who did not complete the experiment to the end were excluded (*n* = 17).

#### Measures and procedure

Measures included the PSIQ, the MCT, and a processing speed measure. The MCT included 48 items, of which 14 were visual, ten were visual-tactile, and 24 were tactile stimuli (similar to those in Study 1), administered in blocks of eight trials (similar to Study 2). The internal consistencies were acceptable for the MCT accuracy, α_cr_ = .63, *r*_SB_ = .65, and high for response latency, α_cr_ = .94, *r*_SB_ = 93.

The processing speed task was conceptualized to run precisely the same as the MCT, with the exception that the comparison pertained to the perceived loudness of two beeps. Accordingly, the processing speed task can be conceived of as requiring similar steps as the MCT, with the key exception that instead of imagery generation, a memory for the loudness of the tone is required. Thus, participants were asked “what is louder [beep1] or [beep2]?” The loudness of the beeps was varied, such that there were five different volume variations, each 2 dB apart, of the same beep of approximately 125 ms in length and was approximately at 380 Hz. The lengths of the beeps were kept short, so that they were clearly audible but required participants’ close attention. The spacing of the beeps followed the exact same procedure as for the ICT, thus occurring at 2 s and 4 s in each trial. If participants took longer than 5 s to respond, the next sound was presented. The internal consistencies were acceptable for processing speed accuracy, α_cr_ = .61, and high for response latency, α_cr_ = .89.

The study was again conducted online, following a near identical procedure to Study 2, whereby participants responded to four demographic questions, completed a 16-item MCT pretest and an 8-item processing speed pretest, whose data were not included in these analyses. Following this, 1-min film clips (as part of a different experimental aim) were played followed by blocks of eight MCT or processing speed items, in a randomized order. Given the confirmatory nature of Study 3, alpha was set to *p* = .01.

### Results and discussion for Study 3

The same winsorizing procedure to Study 2 was adopted for both processing speed and MCT trials, which resulted in winsorizing .15% of responses at 10 s and 1.125% at .35 s. Two items had a response accuracy of less than 75%, one of which was also dropped in Study 2 (softer: fur or stuffed toy, and pointer: needles or nails). Next aggregate scores were created by taking the average response latency and accuracy scores for the processing speed and MCT tasks, by modality. The mean PSIQ imagery rating (scale: 0 = no imagery to 10 = vivid as real life) was also calculated and together these appear in Table 7.

**Table 7:** Descriptive statistics for the MCT, processing speed test, and PSIQ in Study 3

Variable	*M*	*SD*	*N*	Min	Max	Skew	Kurt
Processing speed (acc)	.90	.07	461	.50	1.00	– 1.91	6.65
Processing speed (s)	.87	.26	461	.38	2.83	2.06	8.66
MCT (acc)							
Visual	.94	.08	461	.50	1.00	– 2.27	7.53
Visual-tactile	.93	.09	461	.30	1.00	– 2.11	7.33
Tactile	.92	.08	461	.50	1.00	– 1.59	4.18
MCT (s)							
Visual	1.42	.47	461	.67	4.96	2.35	10.08
Visual-tactile	1.60	.54	461	.79	4.74	2.09	6.92
Tactile	1.55	.50	461	.71	4.50	1.62	4.28
PSIQ	6.74	1.06	448	2.74	9.00	– .38	.13

MCT = mental comparison task, rt = response time/latency, acc = accuracy expressed as a proportion between 0 and 1, PSIQ = Plymouth Sensory Imagery Questionnaire

As can be seen in Table 7, response accuracy was high in both the MCT and processing speed task. Additionally, the processing speed task was answered much more rapidly than the visual modality of the MCT, *d* = 1.44. This suggests that, despite the tasks being precisely matched in terms of demands, the requirement to create two images in the MCT appears to have taken greater processing time, adding face validity to the task. Also, the MCT items in the visual modality were answered more quickly than those in the tactile, *d* = .27, or mixed modality, *d* = 36. Next, correlation coefficients between the imagery and processing speed measures were calculated and these appear in the upper quadrant in Table 8. To test the independent relations between the imagery measures after controlling for processing speed, partial correlations were run, which appear in the lower quadrant of Table 8.

**Table 8:** Correlations and partial correlations between the MCT response latency, processing speed test, and PSIQ in Study 3

	Variable	1	2	3	4	5
1	Processing speed	-	.55*	.52*	.59*	.02
2	MCT visual	-	-	.82*	.84*	.01
3	MCT visual-tactile	-	.75*	-	.82*	.05
4	MCT tactile	-	.78*	.76*	-	.07
5	PSIQ	-	.00	.05	.07	-

Values in the lower quadrant have the influence of processing speed partialed out

MCT = mental comparison task, PSIQ = Plymouth Sensory Imagery Questionnaire

^*^ = *p* < .01

As Table 8 shows, processing speed correlated strongly with the MCT scales, but the MCT scores correlated more strongly still. The partial correlation with processing speed controlled were still strong (≈ .75), indicating that processing speed does not explain MCT performance. Finally, the PSIQ showed little relation with the MCT and processing speed tasks.

The findings from Study 3 replicate and extend those of Study 1 and 2. In terms of replication, it was again found that the self-report imagery scale shows little relation to the MCT. In terms of extension, the findings suggest that the MCT measures more than processing speed for two reasons. Firstly, participants required longer to respond to the MCT than the processing speed task, despite these having closely matched task demands. Second, the MCT scales correlated with each other after partialing out the effect of processing speed.

## General discussion

The primary purpose of this paper was to investigate the extent to which the MCT measures mental imagery as a multimodal construct. Although it is easy to formulate such an aim, it is decidedly difficult to achieve given that mental imagery is not directly observable and has proven very difficult to measure (Pylyshyn, 2002). As reviewed, many mental imagery measures are self-reports (Suica et al., 2022) and focus on vividness (Lacey & Lawson, 2013a). This creates inherent difficulties for capturing between-subject differences and may even index a different aspect of imagery altogether, as discussed below. The current studies found that the MCT resulted in reliable internal consistencies, suggesting that performance was not arbitrary. Second, the response latencies were consistent with processing occurring beyond a mere perceptual or semantic decision level, taking around twice as long as semantic decision tasks (e.g., Siakaluk et al., 2003). Third, processing speed did not explain mental imagery performance. Fourth, although the frequency of the MCT stimuli played a role in predicting response latencies, it did not explain the variance in the models and word length was not a significant predictor. Fifth, inter-modal differences in mental imagery were found, with visual items on the MCT being processed more rapidly and tactile items the slowest. Sixth, some statistically significant relations were found between MCT response accuracy and self-report imagery, providing some evidence for concurrent validity.

Returning to the intermodal differences found on the MCT, this finding could be taken to indicate that the kind of mental imagery measured with the MCT relies on perceptual systems. Of course, this idea is not new and has been proposed by numerous others (Kosslyn et al., 2010), and supported with EEG and fMRI studies (Bird et al., 2010; Pearson, 2019; Yomogida et al., 2004). However, this study adds to previous research because it finds that varying the modality resulted in differences in response latency and accuracy at the behavior level, with vision appearing dominant (Hutmacher, 2019). Thus, it could be that each brain pathway associated with each sensory modality is initially involved in producing a sensorimotor simulation in the form of a mental image. These images may then be categorized and processed, perhaps as advanced perceptual symbols (Barsalou, 1999), during the decision component of the MCT.

Alternatively, it could be that properties of image identification differ across modalities, with for touch requiring temporal information. For instance, research indicates that tactile stimuli are responded to more slowly than visual stimuli (Laasonen et al., 2001), which may transfer to the corresponding mental images. In terms of temporal information, in identifying whether something is rough, a stroke across the stimulus with say one’s fingers is necessary, whereas identifying whether an object is brighter requires comparing an immediate color impression. This would appear to add further validity to the MCT as correspondence between sensory processing and image processing was found. Given that there was a control for word frequency, it is difficult to see how amodal, propositional processing could lead to the observed slower response latencies for tactile stimuli.

The current findings concerning the modest relations between the self-report imagery scales and the MCT require addressing. Given that self-report scales have been validated many times, correlating with each other and showing test–retest reliability and internal consistency (Suica et al., 2022), it would seem difficult to question their psychometric properties. However, as repeatedly argued, it is difficult to see how self-reports of imagery, particularly in light of the belated discovery of aphantasia (Zeman et al., 2015), can be compared between participants. Another idea is that self-report measures of imagery assess explicit mental imagery, whereas MCT performance does not necessarily require explicit, carefully constructed vivid imagery. Arguably, the sensorimotor simulation of the environment is essential for intelligent operating in that environment; hence all people likely have functioning unconscious mental imagery, despite a small percentage claiming no awareness of imagery at all. Relatedly, the self-report measures might have tapped vivid and detailed of imagery, whereas the MCT likely only requires a small amount of image vividness in order to determine the correct response and move on to the next item.

Perhaps, in light of intermodal MCT differences, some people have relative imagery strengths in some modalities but not in others – although the high correlations between modalities on the MCT found are not consistent with this idea. Nevertheless, most people are seemingly unaware that they have more than five senses, having little or no explicit knowledge of the proprioceptive, vestibular, and visceroceptive modalities. Although speculative, it may be that a relative strength could exist in some modalities and a weakness or absence in, say the visual or auditory modalities. Thus, when constructing perceptual representations of the external world, these may be more spatial than visual. Research is therefore needed that expands the MCT and self-report scales to include different modalities, much like the PSIQ does, to then examine phantasic and aphantasic populations.

Turning to the face validity of the MCT, it would appear that this is high. Specifically, it seems difficult to imagine how the MCT could be solved without imagery, although it remains unclear how explicit this needs to be. Specifically, across 126 items (in Study 2), it is unlikely that participants would be able to respond with an accuracy of over 90% to the items based on propositional knowledge. This would require participants to have encountered explicit propositional/declarative formulations regarding each stimulus pair in relation to a sensory property (e.g., that violins are not as shiny as trumpets, generally), or at least to have stored up associations that could lead to reasonable guesses (e.g., trumpet = metal = shiny, violin = wood = dull, therefore trumpet is shinier). Such a string of extrapolations would likely require more time than the 1.2-s response latency observed in Study 2 and would also be difficult to solve with the high accuracy rate also observed here. Further, as discussed, it is difficult to see how this way of solving the tasks would lead to the longer response latencies for the non-visual stimuli. Accordingly, it seems reasonable to assume that at some level, whether conscious or not, mental image comparisons in the form of sensorimotor simulations were being made.

## Limitations

The stimulus pairs in the MCT task were not presented in a counter-balanced order (e.g., word A always came before word B for every participant), although it was ensured that the first and second stimuli were equally represented as the correct response. Accordingly, it may be possible that participants guessed the correct response before the second stimulus had been presented in some instances. Given that the MCT requires a comparison between two stimuli on a particular sensory property, this guessing effect is likely only in extreme instances where there is unlikely to be something, for instance, pointier still than a needle. Presumably, this limitation might have led to shorter response latencies for such items, adding to the variance.

The current study is somewhat limited by the lack of a validation instrument for the MCT. Measures of vividness were used, which although providing a face-valid way to estimate imagery via introspection, these tend to overly rely on vividness (Lacey & Lawson, 2013a), which is not so accessible to implicit processing. Such an instrument would need to capture between-subject differences and be performance based. One possibility is the mental rotation task, however, as outlined, it is not clear to what extent this task involves non-visual imagery. Other options might include measures of auditory imagery (Hubbard, 2010), taste comparison tasks (Bensafi et al., 2013) or visual imagery, such as tasks not yet standardized, such as counting the number of windows in one’s house, the number of letters in the alphabet with an arch etc. Additionally, the MCT could be validated using neuropsychological methods, to determine which perceptual and executive systems are involved at different points of processing.

At a broader, experiential level, mental imagery often involves aesthetic contemplation of scenes “painted” or relived before one’s mind (e.g., Marks, 1999). Rapid responses to questions on whether a needle is pointer than a nail seem a fair distance away from richer image construction and inspection. Although it is likely that having faster access to the more basic perceptual symbols measured with the MCT might facilitate complex scene imagery, this relation needs further examination. Subsequently, more work is needed that looks at measuring imagery quality.

Findings are broadly consistent with the four aspects of mental imagery outlined, namely that imagery is multimodal (Lacey & Lawson, 2013b), overlaps with perception (Addis, 2020; Kosslyn et al., 2010), can be unconscious (Brogaard, & Gatzia, 2017), and may indeed be epiphenomenal (Cole et al., 2022; Pylyshyn, 2002). However, to test the last two propositions, research is required in which self-reported hyperphantasics, aphantasics, and phantasics are tested on both self-report scales of imagery and the MCT, alongside cognitive tasks. However, an interesting question remains as to whether experiencing mental imagery is causally beneficial for cognitive processing.

## Conclusion and outlook

The purpose of the current study was to develop and propose a behavioral measure of mental imagery that is easy to administer to a range of samples and is multimodal. The MCT showed good internal consistency, was sensitive to modality differences in processing, showed some links with self-report scales, and explained variance in imagery over and above processing speed. The hope is that the MCT can spark renewed research into mental imagery now that another instrument outside of self-report imagery scales exists. Many avenues to mental imagery research exist, from further developing and validating the MCT, investigating the role of the MCT in cognitive processing and learning, and testing how educational experiences might affect mental imagery.

## Data Availability

Data can be accessed: https://osf.io/vf27z/?view_only=43c4db49648f428c914ebdcc4e191f27.

## Appendix: Mental comparisons task items from study 2

**Table:**

Property	(translation)	Word A	(Translation)	Word B	(Translation)
heller	lighter	Schokolade	Chocolate	Ei	Egg
heller	lighter	schwarz	Black	gelb	Yellow
heller	lighter	Leder	Leather	Taschentuch	Handkerchief
heller	lighter	Kaese	Cheese	Salami	Salami
heller	lighter	Sonnenblume	Sunflower	Baum	Tree
heller	lighter	Papier	Paper	Pappe	Cardboard
heller	lighter	Reis	Rice	Brot	Bread
heller	lighter	Pfeffer	Pepper	Salz	Salt
heller	lighter	Matsch	Mud	Marmor	Marble
heller	lighter	Kakao	Cocoa	Mehl	Flour
heller	lighter	Cola	Cola	Limonade	Lemonade
heller	lighter	Schaf	Sheep	Elefant	Elephant
heller	lighter	Birne	Pear	Pflaume	Plum
heller	lighter	Limette	Lime	Kiwi	Kiwifruit
heller	lighter	Wal	Whale	Eisbaer	Polar bear
heller	lighter	Baer	Bear	Schwan	Swan
heller	lighter	Erde	Earth	Diamant	Diamond
*heller*	*lighter*	*Feuer*	*Fire*	*Wasser*	*Water*
heller	lighter	TipEx	Twink	Edding	Marker pen
heller	lighter	Blitz	Lightning	Abendrot	Evening
heller	lighter	Nacht	Night	Tag	Day
heller	lighter	Eisbaer	Polar bear	Pinguin	Penguin
heller	lighter	Sahne	Cream	Saft	Juice
heller	lighter	Kohle	Coal	Gurke	Cucumber
heller	lighter	Pfirsich	Peach	Kirsche	Cherry
heller	lighter	Wiese	Meadow	Wald	Forest
heller	lighter	Schnee	Snow	Gras	Grass
heller	lighter	Milch	Milk	Kaffee	Coffee
heller	lighter	braun	Brown	weiss	White
heller	lighter	Biber	Beaver	Eisbaer	Polar bear
heller	lighter	Zitrone	Lemon	Tomate	Tomato
heller	lighter	Strand	Beach	Meer	Sea
heller	lighter	Asche	Ash	Kerze	Candle
heller	lighter	Apfelschorle	Apple juice	Traubenschorle	Grape juice
heller	lighter	Tafel	Chalkboard	Kreide	Chalk
heller	lighter	Sand	Sand	Schlamm	Mud
heller	lighter	Taube	Dove	Ente	Duck
heller	lighter	Spezi	Cola	Schaum	Foam
heller	lighter	Kaesesosse	Cheese sauce	Tomatensosse	Ketchup
heller	lighter	Silberkette	Silver chain	Goldkette	Gold chain
heller	lighter	Schokoeis	Chocolate ice-cream	Vanilleeis	Vanilla ice-cream
heller	lighter	Finger	Fingers	Reife	Tire
lauter	louder	Tuerknallen	Door-slam	Gewehrschuss	Gunshot
lauter	louder	Kamm	Comb	Foehn	Hairdryer
lauter	louder	Gitarre	Guitar	Schlagzeug	Drums
lauter	louder	Triangel	Triangle	Trommel	Drum
lauter	louder	Hubschrauber	Helicopter	Zug	Train
lauter	louder	Explosion	Explosion	Flamme	Flame
lauter	louder	Sprechen	Speaking	Schreiben	Writing
lauter	louder	Stechbeitel	Chisel	Motorsaege	Power saw
lauter	louder	Vogelgezwitscher	Chirping	Hundebellen	Barking
lauter	louder	Blinker	Indicator	Hupe	Horn
lauter	louder	Rasenmaeher	Lawnmower	Gartenschlauch	Garden hose
lauter	louder	Auto	Car	Inliner	Inliner
lauter	louder	Schwein	Pig	Schlange	Snake
lauter	louder	Regen	Rain	Spruehnebel	Mist
lauter	louder	Wasserfall	Waterfall	Binnensee	Lake
lauter	louder	Tacker	Stapler	Kugelschreiber	Pen
lauter	louder	Mixer	Mixer	Schneebesen	Whisk
lauter	louder	Decke	Blanket	Wecker	Alarm
lauter	louder	Henne	Hen	Hahn	Rooster
lauter	louder	Fluestern	Whispering	Schreien	Shouting
lauter	louder	Wasserkocher	Kettle	Toaster	Toaster
lauter	louder	Geiger	Violinist	Fliege	Fly
lauter	louder	Sirene	Siren	Tuerklingel	Doorbell
lauter	louder	Schraubenzieher	Screwdriver	Hammer	Hammer
lauter	louder	Kirchglocke	Church bell	Fahrradklingel	Bicycle bell
lauter	louder	Kissen	Pillow	Chipstuete	Chip bag
lauter	louder	Fisch	Fish	Vogel	Bird
*lauter*	*louder*	*Rassel*	*Rattle*	*Pfeife*	*Whistle*
lauter	louder	Presslufthammer	Jackhammer	Bohrmaschine	Drill
lauter	louder	Esel	Donkey	Maus	Mouse
lauter	louder	Donner	Thunder	Wind	Wind
lauter	louder	Trompete	Trumpet	Harfe	Harp
lauter	louder	Heissluftballon	Hot air balloon	Dampflock	Steam train
lauter	louder	Katze	Cat	Hund	Dog
lauter	louder	Schneeregen	Sleet	Hagelkoerner	Hailstone
lauter	louder	Meeresrauschen	Waves lapping	Orkan	Hurricane
lauter	louder	Rasiergeraet	Razor	Schere	Scissors
lauter	louder	Dudelsach	Bagpipes	Floete	Flute
lauter	louder	Joggen	Jogging	Spazieren	Walking
lauter	louder	Highheels	High heels	Hausschuh	Slipper
lauter	louder	Kuehlschrank	Refrigerator	Waschmaschine	Washing machine
lauter	louder	Bus	Bus	Flugzeug	Airplane
kratziger	scratchier	Grashalm	Grass blade	Tannenzweig	Firtree needle
kratziger	scratchier	Buerste	Brush	Schwamm	Sponge
kratziger	scratchier	Teppich	Carpet	Bettdecke	Duvet
kratziger	scratchier	Gras	Grass	Heu	Hay
kratziger	scratchier	Bart	Beard	Haare	Hair
kratziger	scratchier	Feile	File	Seide	Silk
*kratziger*	*scratchier*	*Taschentuch*	*Handkerchief*	*Wolle*	*Wool*
kratziger	scratchier	Watte	Cotton wool	Igel	Hedgehog
kratziger	scratchier	Zahnseide	Dental floss	Zahnbuerste	Toothbrush
kratziger	scratchier	THemd	Tee-shirt	Pullover	Sweater
kratziger	scratchier	Schleifpapier	Sandpaper	Bastelkarton	Cardboard
kratziger	scratchier	Malerpinsel	Paintbrush	Kosmetikpinsel	Cosmetic brush
kratziger	scratchier	Dornenbusch	Thorn	Rosenbluete	Petal
kratziger	scratchier	Fels	Rock	Fliese	Tile
weicher	softer	Polster	Cushion	Kissen	Pillow
weicher	softer	Moos	Moss	Rinde	Bark
weicher	softer	Kuchen	Cake	Brot	Bread
weicher	softer	Zwieback	Rusk	Semmel	Bread roll
weicher	softer	Flummi	Rubber ball	Murmel	Marble
weicher	softer	Haut	Skin	Feder	Feather
weicher	softer	Bauch	Belly	Kopf	Head
weicher	softer	Radiergummi	Eraser	Buchruecken	Book spine
weicher	softer	Apfel	Apple	Banane	Banana
weicher	softer	Sofa	Sofa	Stuhl	Chair
weicher	softer	Pfirsich	Peach	Zucchini	Zucchini
weicher	softer	Butter	Butter	Kaese	Cheese
weicher	softer	Rolladen	Shutter	Vorhang	Curtain
weicher	softer	Zahn	Tooth	Handschuh	Glove
weicher	softer	Knochen	Bone	Muskel	Muscle
weicher	softer	Nuss	Nut	Erdbeere	Strawberry
*weicher*	*softer*	*Stofftier*	*Stuffed toy*	*Fell*	*Fur*
weicher	softer	Wange	Cheek	Knie	Knee
weicher	softer	Beton	Concrete	Holz	Wood
weicher	softer	Stahl	Steel	Styropor	Styrofoam
weicher	softer	Baguette	Baguette	Roggenbrot	Ryebread
*weicher*	*softer*	*Hausschuh*	*Slipper*	*FlipFlop*	*Flipflop*
weicher	softer	Kartoffelbrei	Mashed potatoes	Pfannenkuchen	Pancake
*weicher*	*softer*	*Roulade*	*Roulade*	*Toast*	*Toast*
weicher	softer	Schokolade	Chocolate	Gummibaer	Gummi bear
weicher	softer	Gips	Plaster	Knete	Dough
weicher	softer	Steinkugel	Stone	Kuerbis	Pumpkin
weicher	softer	Gartenbank	Garden bench	Luftmatratze	Air mattress

Italicized items were dropped from the analysis because the response accuracy was below 75% for these

## Code availability

Code for the analyses can be accessed: https://osf.io/vf27z/?view_only=43c4db49648f428c914ebdcc4e191f27.
